# Swine Is a Possible Source of Hepatitis E Virus Infection by Comparative Study of Hepatitis A and E Seroprevalence in Thailand

**DOI:** 10.1371/journal.pone.0126184

**Published:** 2015-04-30

**Authors:** Pattaratida Sa-nguanmoo, Nawarat Posuwan, Preeyaporn Vichaiwattana, Norra Wutthiratkowit, Somchai Owatanapanich, Rujipat Wasitthankasem, Thanunrat Thongmee, Kittiyod Poovorawan, Apiradee Theamboonlers, Sompong Vongpunsawad, Yong Poovorawan

**Affiliations:** 1 Center of Excellence in Clinical Virology, Department of Pediatrics, Faculty of Medicine, Chulalongkorn University, Bangkok, Thailand; 2 Narathiwat Ratchanakarin Hospital, Bang Nak, Narathiwat, Thailand; 3 King Narai Hospital, Khao Sam Yot, Lop Buri, Thailand; 4 Department of Clinical Tropical Medicine, Faculty of Tropical Medicine, Mahidol University, Bangkok, Thailand; University of Minnesota, UNITED STATES

## Abstract

Hepatitis A virus (HAV) and hepatitis E virus (HEV) infection in developing countries are associated with contaminated food or water. Although Thailand is non-endemic for HEV, sporadic infections may occur from zoonotic transmission. Individuals between 7 months to 69 years (mean age = 32.8) from predominantly Islamic Narathiwat (n = 305) and swine farm-dense Lop Buri (n = 416) provinces were screened for anti-HEV and anti-HAV antibodies by commercial enzyme-linked immunosorbent assay and automated chemiluminescent microparticle immunoassay, respectively. Seroprevalence and relative antibody titers were analyzed according to age groups. HAV IgG antibody positive rates in Lop Buri and Narathiwat residents were 39.9% and 58%, respectively (*p* < 0.001). Greater than 90% of individuals >50 years old in both provinces possessed anti-HAV IgG. In contrast, seroprevalence for anti-HEV IgG was much higher in Lop Buri (37.3%) than in Narathiwat (8.9%) (*p *< 0.001). Highest anti-HEV IgG prevalence was found among 21-30 year-olds (50%) in Lop Buri and 41-50 year-olds (14.1%) in Narathiwat. In summary, fewer individuals possessed anti-HEV IgG in Narathiwat where most residents abstained from pork and fewer swine farms are present. Therefore, an increased anti-HEV IgG seroprevalence was associated with the density of swine farm and possibly pork consumption. Adults were more likely than children to have antibodies to both HEV and HAV.

## Introduction

Hepatitis E virus (HEV) is a non-enveloped positive-sense RNA virus and the sole member of the genus *Hepevirus* in the family *Hepeviridae*. HEV infection had been associated with poor sanitation and unsafe drinking water. Each year approximately 20 million individuals are infected, resulting in approximately 56,000 deaths [1]. HEV infection is generally self-limiting but may cause acute liver failure with low mortality among healthy individuals but significantly higher mortality in pregnant women [2]. Symptoms can include fever, nausea, vomiting, abdominal pain and jaundice [3]. Although there are four HEV genotypes, only one serotype exist [4]. Genotypes 1 and 2 are often found in developing countries and infect only humans, while genotypes 3 and 4 are found in both humans and animals and have been identified in developed countries [5].

Like HEV, hepatitis A virus (HAV) is transmitted by contaminated food or water and is often associated with poor sanitation. In addition, HAV can be transmitted through close-contact with an infectious person. It is also a non-enveloped positive-sense RNA virus belonging to the family *Picornaviridae* in the genus *Hepatovirus*. There are 6 genotypes of HAV (designated I to VI) in which genotype I, II, and III have been identified in human [6]. Like HEV, there is only one HAV serotype [4]. Although several effective vaccines are available and HAV infection is self-limiting, HAV still affects 1.4 million individuals annually [7]. HAV infection of children in developing countries is generally asymptomatic and provides immunity from reinfection during adulthood [8]. However, HAV outbreaks do occur in industrialized countries as a result of contaminated produce including green onions, tomatoes and berries [9,10].

Although HEV is commonly transmitted via fecal-oral route in endemic regions, autochthonous HEV infections in developed countries are increasingly recognized [11]. HEV infection can be problematic in the immunocompromised as a result of organ transplantation [12] or blood transfusion [13]. Furthermore, HEV can be acquired from the consumption of pork liver food products [14]. Reverse-transcription polymerase chain reaction (RT-PCR) for viral RNA is useful in detecting active infection and the presence of HEV-specific immunoglobulins can indicate past exposure to HEV. Anti-HEV IgM can persist for several months and decline after resolution from infection, but anti-HEV IgG remains detectable for years [15].

Previous studies have shown that urban residents and occupations requiring higher educations are associated with lower seroprevalence of HEV as these factors may be surrogates for better sanitation and hygiene [16, 17]. In addition, evidence linking the consumption of pork products and increased risk for HEV infection is of particular concern [11]. In Southeast Asia, few studies have examined the association between HEV seroprevalence and either consumption of pork or occupational exposure to pigs. We therefore examined anti-HEV antibodies in two similar-sized yet demographically different provinces of Thailand where swine farm densities are different due to local norms. We also assessed the prevalence of antibodies against HAV in these communities for comparison.

## Materials and Methods

This study was approved by the Institutional Review Board of the Faculty of Medicine, Chulalongkorn University (IRB No. 377/57) followed the Helsinki Declaration on medical research. Written informed consent was obtained from study participants or their parents and data such as age, gender and address was collected. Serum samples were analyzed at the Center of Excellence in Clinical Virology, Department of Pediatrics, Faculty of Medicine, Chulalongkorn University. All samples were treated as anonymous.

### Study area and population

**Fig 1:**
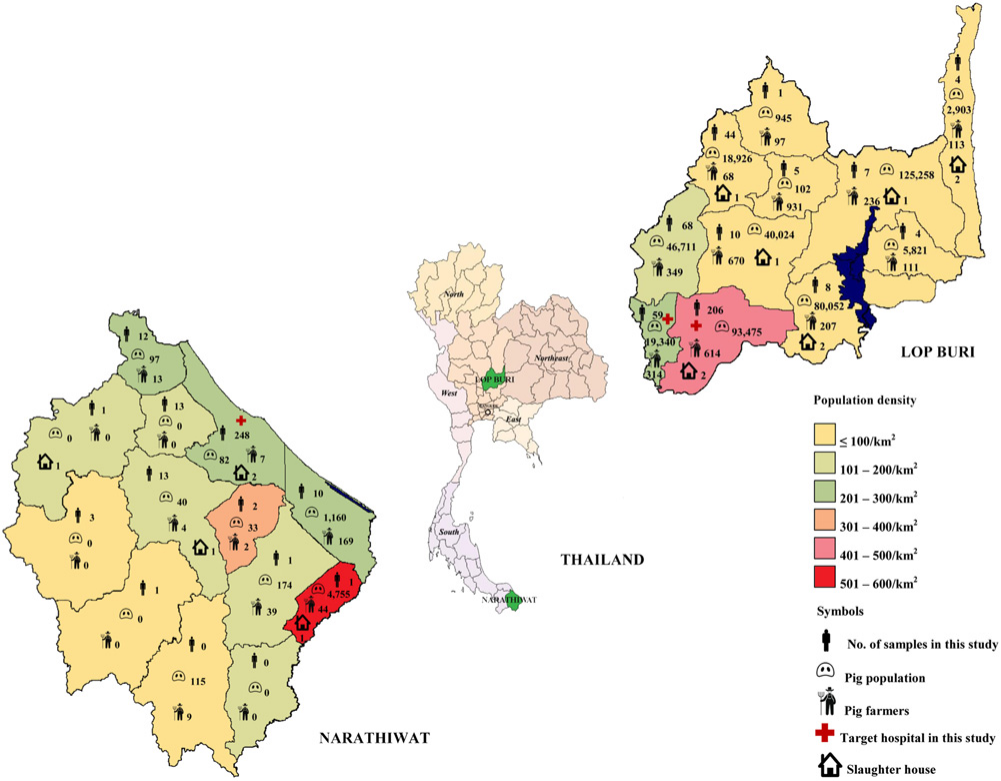
Geographical and population characteristics in the provinces of Lop Buri and Narathiwat. Map indicates the locations of Lop Buri and Narathiwat with information on their respective population density. The number of samples from individuals residing in each district is indicated on the map. Approximate pig population, pig farmers, and slaughter houses in each district are noted.

The subjects were recruited between March 1 and October 31, 2014 during a separate hospital-based, cross-sectional national study on the impact of hepatitis A, B, and C. Lop Buri and Narathiwat were chosen as target sites for central and southern part of Thailand, respectively (Fig 1). Individuals between 6 months to 60 years were healthy volunteers or patients who attended pediatrics or general medicine clinics with no known immunological or chronic conditions. A total of 721 individual samples (311 males, 410 females; mean age 32.8 ± 17.0) were obtained from residents residing in Lob Buri and Narathiwat. Samples from 416 individuals (214 males, 202 females) between the ages of 7 months to 69 years (mean age 32.8 ± 17.2) were obtained from King Narai Hospital and Tha Wung district hospital in Lop Buri. Samples from 305 individuals (97 males, 208 females) between the ages of 3–59 years (mean age 32.8 ± 16.8) were obtained from Narathiwat Ratchanakarin Hospital and represented all but two districts (Sukhirin and Waeng) of Narathiwat.

### Seroprevalence assay

Serum samples were analyzed for HEV IgG antibody using 96-well plate ELISA (Anti-Hepatitis E Virus ELISA IgG, Euroimmun, Lübeck, Germany) according to manufacturer’s instructions. The limit of detection for the anti-HEV IgG test was 0.1 IU/mL. HAV IgG antibodies were measured by automated chemiluminescent microparticle immunoassay (ARCHITECT HAVab-IgG, Abbott Laboratories, Abbott Park, IL) which utilized HAV-coated paramagnetic microparticles to detect anti-HAV IgG. The resulting chemiluminescent reaction was measured as relative light units (RLUs) compared to cutoff signal (S/CO). Samples with S/CO values > 1.00 were considered reactive for IgG anti-HAV, while specimens with S/CO values < 1.00 were considered nonreactive.

### Data analysis

Gender and anti-HEV or anti-HAV antibody results were recorded as frequency and percentage with mean age and standard deviations (SD). Chi-square test provided comparison between groups of categorical variables. Student’s t-test provided comparison between groups of continuous variables. The analyses were performed on SPSS version 17.0 (SPSS Inc., Chicago, IL).

## Results

### Study population of Lop Buri and Narathiwat

Thailand is located in Southeast Asia adjacent to neighboring Myanmar, Laos, Cambodia, and Malaysia. With a population of ~63 million, the majority of Thais practices Buddhism (94.6%) and Islam (4.6%), of which the latter predominates in southern Thailand. Thailand specializes in agriculture and livestock-based farming including pork production. Large-scale swine farms are primarily located in central Thailand (~57%), while very few small and mostly family farms are located in the south [18] (Fig 1).

Although Lop Buri and Narathiwat have comparable population size, there are differences in demographics and socio-economic differences (Table 1). Lop Buri is a central province ~154 km from the capital Bangkok. The total population is ~756,127 and predominantly Buddhist (99.8%) [19]. There are estimated 434,386 animals and 2,881 farmers, some on commercial scale. This density is relatively high given that there are swine farms in nearly all provincial districts [20,21].

**Table 1:** Comparison of socio-economic data between Lop Buri and Narathiwat.

		Lop Buri^a^ ^,^ ^b^ ^,^ ^c^ ^,^ ^d^ ^,^ ^e^	Narathiwat^a^ ^,^ ^b^ ^,^ ^f^ ^,^ ^g^ ^,^ ^h^
Area		6,200 km^2^	4,475 km^2^
Population		756,127	766,145
Population density		120/km^2^	170/km^2^
Religion	Buddhism	99.76%	17.0%
	Christianity	0.11%	1.0%
	Islam	0.13%	83.0%
Education	No education	5.84%	4.87%
	Less than elementary level	3.78%	4.49%
	Elementary level	48.59%	56.94%
	Lower secondary education	16.58%	14.44%
	Upper secondary level (general)	9.81%	9.91%
	Upper secondary level (vocational)	4.06%	0.81%
Occupation	Agricultural and fishery workers	29.04%	33.75%
	Service provider and seller	35.78%	22.85%
Income Per Capita (Baht)		95,412	71,786
Health system	Hospital (total bed)	16 (1,769)	13 (1,040)
	Health care worker	2,705	1,853
Pig population		434,386	6,456
Pig farmers		2,881	287

^a^National Statistical Office;

^b^Information Technology and Vocational Manpower Center;

^c^Lop Buri Governor’s Office;

^d^Lop Buri Provincial Public Health Office;

^e^Lopburi Provincial Livestock Office;

^f^Narathiwat National Statistical Office;

^g^Narathiwat Provincial Public Health Office;

^h^Narathiwat Provincial Livestock Office.

In contrast, the southern-most province of Narathiwat is located immediately north of Malaysia. The total population is ~762,622 and most residents are Muslim Thais (83%). There are an estimated 287 farmers who keep 6,456 animals, mostly for local consumption by non-Muslims [22].

### Anti-HEV antibody prevalence and titers

**Fig 2:**
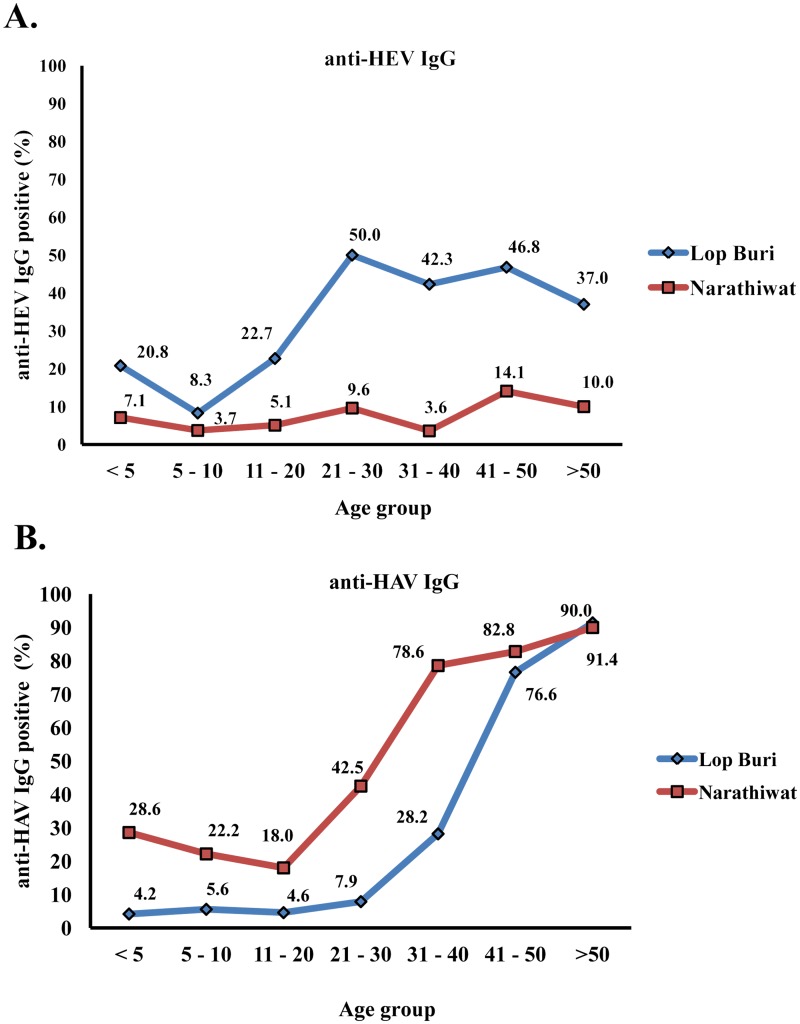
Distribution of seroprevalence for Lop Buri and Narathiwat by age group. (A) anti-HEV IgG antibody and (B) anti-HAV IgG antibody prevalence in different age groups.

We found that the overall anti-HEV IgG positive rate was 37.3% in Lop Buri and 8.9% in Narathiwat (*p* <0.001) (Table 2). Mean age of individuals with positive anti-HEV antibody was 36.4 ± 14.6 in Lop Buri and 38.1 ± 15.9 in Narathiwat (*p* = 0.58). Although the anti-HEV IgG positive rates were generally below 50.0%, seroprevalence in Lop Buri was much higher than Narathiwat (37.3% VS 8.9%; *p* < 0.001) especially among adults (Fig 2A).

**Table 2:** Anti-HEV and anti-HAV IgG seropositive rates in Lop Buri and Narathiwat population associated with gender and age group.

	Total subjects	Mean age	No. of test		anti-HEV IgG positive (%)			anti-HAV IgG positive (%)		
			Lop Buri	Narathiwat	Lop Buri	Narathiwat	*p*-value^a^	Lopburi	Narathiwat	*p*-value^a^
Sex										
Male	311		214	97	37.9	8.3	<0.001	37.4	56.7	0.002
Female	410		202	208	36.6	9.1	<0.001	41.1	58.7	0.001
Age										
<5	38	2.5±1.2	24	14	20.8	7.1		4.2	28.6	0.031
5–10	63	7.3±1.8	36	27	8.3	3.7		5.6	22.2	0.049
11–20	83	15.8±3.3	44	39	22.7	5.1	0.023	4.6	18.0	
21–30	149	25.7±2.6	76	73	50.0	9.6	<0.001	7.9	42.5	<0.001
31–40	106	35.2±2.8	78	28	42.3	3.6	<0.001	28.2	78.6	<0.001
41–50	141	45.4±2.9	77	64	46.8	14.1	<0.001	76.6	82.8	
>50	141	55.5±3.2	81	60	37.0	10.0	<0.001	91.4	90.0	
**Total**	**721**	**32.8±17.0**	**416**	**305**	**37.3**	**8.9**	**<0.001**	**39.9**	**58.0**	**<0.001**

^a^ *p*-value <0.05 denotes statistical significance.

When individuals were categorized into 7 age groups, highest seroprevalence was observed among those between 21–30 in Lop Buri (50.0%) and 41–50 (14.1%) in Narathiwat. Age group with the lowest prevalence of anti-HEV IgG was 5–10 year-olds in Lop Buri (8.3%) and 31–40 year-olds in Narathiwat (3.6%). The overall anti-HEV titers among the different age groups were similar in both provinces (4.4±5.7 IU/mL in Lop Buri and 4.4±4.7 IU/mL in Narathiwat; *p* = 0.992).

### Anti-HAV antibody prevalence and titers

For comparison, we also aimed to assess the seroprevalence of HAV, another food borne virus that is endemic in Thailand. We found that the overall anti-HAV antibody was 39.9% in Lop Buri and 58.0% in Narathiwat (*p* < 0.001). Seropositive rates were similar between men and women in both Lop Buri (41.1% versus 38.8%; *p* = 0.69) and in Narathiwat (58.7% versus 56.7%; *p* = 0.80). The mean age of individuals with positive anti-HAV antibody was significantly different between Lop Buri (47.1 ± 10.9) and Narathiwat (40.6 ± 14.5) (*p* < 0.001). Moreover, seropositive rate of anti-HAV IgG was consistently higher among Narathiwat residents in all age groups compared to residents of Lop Buri (Fig 2B). The presence of anti-HAV for both provinces was highest among individuals >50 years in which ≥ 90% tested positive.

The overall S/CO titers of anti-HAV IgG antibody in residents of both provinces were slightly different but not statistically significant (12.4±2.8 in Lop Buri and 13.0±3.4 in Narathiwat; *p* = 0.051). Furthermore, there were no significant differences in the anti-HAV titers among different age groups.

## Discussion

HEV infection from porcine zoonosis has long been suspected of contributing to the entero-transmissible form of hepatitis similar to that caused by HAV [23]. In addition, occupational exposure to pigs has been linked to HEV infection [24]. In this study, the exposure risk to HEV was examined from a different perspective using religion as a surrogate for evaluating disease risk. We hypothesized that the likelihood of detecting HEV antibodies in individuals from two similarly sized regions may differ based on diet, religious and social norms. We found that although both HEV and HAV are foodborne, the seroprevalence of anti-HAV IgG was significantly higher in Narathiwat than in Lop Buri. In contrast, relatively low seroprevalence (3–14%) for anti-HEV IgG among individuals of different age groups in Narathiwat contrasted with higher seropositive rates (8–50%) found among Lop Buri residents.

By studying the prevalence of HEV and HAV, which share the same mode of transmission, one might expect to detect similar frequency of antibodies to these viruses among Thais. Both HAV and HEV are foodborne, why then was the presence of HAV antibodies more prevalent in individuals living in Narathiwat, yet HEV seroprevalence was significantly lower? One possible answer might be the zoonotic transmission of HEV through occupational contact with swine and/or pork consumption. Buddhists comprising the majority of residents in Lop Buri have no dietary restrictions of pork products and therefore do raise swine in the farm or work in pork-processing facilities. These individuals may be exposed to HEV as swine farmers, animal transporters, abattoir workers, pork handlers, or consumers. Moreover, this province is the largest pork producer in central Thailand. Of the 226 swine farms in Lop Buri, most are large facilities that process and distribute meat to neighboring provinces including pork sold in metropolitan Bangkok. These factors may therefore increase the exposure of HEV in the Lop Buri population. In contrast, most Narathiwat residents adhere to Islam, which proscribes the consumption of pork. Persons of Islamic faith also do not engage in activities involving swine raising, butchering, and selling of pork products. As a result, individuals in Narathiwat are at a decreased risk of HEV infection. It is interesting to note that contact with swine was a risk factor for Nipah virus infection in the predominantly Islamic Malaysia when researchers observed that infected individuals were all non-Muslims [25]. Finally, evidence from comparative studies between HEV sequences isolated in swine and human strongly support zoonotic transmission in Europe such as Belgium [26], France [14], and the UK [27].

Findings from this study reaffirmed evidence that the prevalence of HEV IgG antibodies were higher among older men than other groups [11, 17]. It is not known whether being older is a risk factor for HEV seroprevalence or that having lived through certain years during times of outbreak is a risk factor as was identified in England [28]. No particular patterns can be discerned from the levels of antibody titers against HAV or HEV to suggest recent viral infection in our cohorts. It is noteworthy that higher HAV and HEV seroprevalence observed among those <5 years old found in this study was not unexpected. Transplacental transfer of anti-HAV antibodies from mothers to infants may account for some of the higher seroprevalence in young children [29]. Furthermore, relatively high seroprevalence of anti-HAV antibody in Thailand among those ≤ 2 years old (between 3%–23% depending on the province surveyed) had previously been documented [30].

Immunity to HEV is not life-long as serum anti-HEV IgG does decline several years after initial infection [31]. The duration of IgG immunity can last as long as 12 years after acute infection, but reinfection has been documented and therefore immunity to HEV may be limited [11]. The waning of HEV IgG over an individual’s lifetime is consistent with our observed decreased in seroprevalence among those >50 years old, especially in the Lop Buri cohort.

Previous efforts to determine the burden of HEV infection in Thailand using HEV seroprevalence survey found 23.3% in occupational high-risk group and 6.5% in ethnic Hmong population in the northern Thailand [16, 32]. The overall seroprevalence of 37% found in this study was higher than the 9–22% [33] from a previous Thai study, which may be due to better detection sensitivity. It was also higher than the 14% from another study, which only surveyed Thai men between the ages 18–30 [34]. Globally, prevalence of anti-HEV IgG varies and previous studies conducted in the general population included mostly adults. For example, the overall HEV seroprevalence was 5.9% in Korea [24], 4.6%–6.7% in Japan [35], and 17% in Germany [36]. Among blood donors, the rate of HEV antibodies found were 4.9% in Switzerland [37], and 3.7% in Japan [38].

Detection of HAV antibodies generally indicate past infection and its presence is generally recognized as lifelong [8]. Prior to 1980, >97% of Thais ≥16 years old tested positive for anti-HAV [39]. Improved sanitation and hygiene over the past three decades, however, have helped to reduce exposure to HAV infection and thus HAV immunity in the general population. As a result, sporadic outbreaks in recent years have rendered young Thais vulnerable to HAV infection [40]. Our previous assessment of the overall anti-HAV seroprevalence in the Thai population was 27%, but it was as high as 71% among those residing in regions bordering Myanmar [41]. Similarly, higher HAV seroprevalence in Narathiwat may result from reduced access to better sanitation or exposure to unsafe drinking water or contaminated produce. Consistent with this observation is the many documented outbreaks of HAV in southern Thailand including Narathiwat [42–44].

Hepatitis E vaccine has been tested and is licensed for use in China [45]. Despite the availability of an effective HAV vaccine [46], universal HAV vaccination in the general Thai population is currently not recommended based on cost-effective analysis [47]. In addition, HAV vaccine is not included in the vaccination schedule of most EU countries [10].

There are several limitations in this study. There were fewer men than women and fewer residents from Narathiwat than from Lop Buri in this seroprevalence survey. The seroprevalence of individuals living in these two provinces may or may not reflect the immunity to either HAV or HEV in central and southern Thailand. Furthermore, information on the occupation or religion of individuals would have been valuable. We were unable to ascertain whether pre-existing antibodies surveyed in this study resulted from recent or distant infection or whether they were specific for particular genotypes, although substantial evidence suggests that HEV genotype 3 may be the predominant strain circulating in Thailand [48–53]. Since ELISA results may differ depending on the commercial source, definitive determination of past HEV exposure may benefit from confirmation via HEV-specific cell-mediated immunity with interferon-gamma ELISPOT assay [54].

The prevalence of HEV in pigs and sequence comparison with strains isolated in Thais will provide better understanding into transmission and viral co-evolution with the porcine and human hosts. Evidence-based knowledge should facilitate policy-making decisions towards the improvement of hygienic practices, infrastructure for agribusiness, and management of enteric viral infections in the region.
